# Clinical practice guidelines for acute kidney injury: a systematic review of the methodological quality

**DOI:** 10.3389/fmed.2025.1567359

**Published:** 2025-07-24

**Authors:** Ruizhe Li, Lanxia Wu, Xinhui Wu, Qingyang Fu, Mingyu Xu, Xu Zhai, Junfei Chen

**Affiliations:** ^1^Department of Cardiology, Peking University Third Hospital, Beijing University, Beijing, China; ^2^School of Psychology, Shandong Second Medical University, Weifang, China; ^3^Department of Nephrology, Shandong Electric Power Central Hospital, Jinan, China; ^4^Department of Orthopedics, Qilu Hospital of Shandong University, Cheeloo College of Medicine, Shandong University, Jinan, China; ^5^Department of Pediatric Surgery, Qilu Hospital of Shandong University, Cheeloo College of Medicine, Shandong University, Jinan, China

**Keywords:** systematic review, clinical practice guidelines, evidence-based, acute renal failure, acute kidney injury

## Abstract

**Objective:**

Hospitalized patients, particularly those in the ICU, face a significant risk of acute kidney injury (AKI). Clinical practice guidelines (CPGs) that outline diagnostic and therapeutic strategies for AKI can be valuable tools for healthcare professionals, but their effectiveness hinges on their quality. The research aims to conduct a systematic review focusing on the methodological and reporting quality of CPGs addressing acute kidney injury.

**Design:**

A systematic review of CPGs for the management of acute kidney injury in adult patients.

**Data resources:**

PubMed and other related databases were systematically searched from January 2012 to January 2023 to collect clinical guidelines and expert consensus on acute kidney injury. After summarizing the basic information, the methodological and reporting quality of the included guidelines or expert consensus was evaluated using the AGREE II and RIGHT tools.

**Results:**

A total of 8 CPGs from 6 countries and regions were included in this study, and the period was 2012–2023. In terms of methodological quality, 1 guideline was a level strongly recommended, and 6 were level B, generally recommended guidelines. While the evaluated guidelines demonstrated moderate overall reporting completeness, critical deficiencies persist in contextual transparency and quality assurance protocols, necessitating prioritized remediation in future iterations.

**Conclusion:**

Our appraisal identifies the 2012 KDIGO guideline as the highest-quality AKI guidance framework currently available, which has driven global standardization of diagnostic criteria and management protocols. Our analysis reveals critical gaps in the practical applicability and transparency of current clinical practice guidelines, particularly regarding implementation strategies tailored to different populations, such as aging populations. Future iterations must prioritize demographic inclusivity, with explicit recommendations addressing the diagnostic challenges and management complexities of AKI among different populations.

## Introduction

Acute kidney injury (AKI), formerly termed acute renal failure (ARF), is defined as an abrupt and sustained decline in kidney function. This condition manifests clinically with symptoms such as azotemia, disturbances in water-electrolyte balance, and systemic manifestations (1). AKI represents a prevalent and life-threatening clinical entity affecting both general ward admissions and critically ill patients in intensive care units (ICUs). The incidence of AKI is estimated to affect approximately 20% of general ward patients and 50% of intensive care unit patients (2, 3). Furthermore, the mortality rate for AKI patients requiring renal replacement therapy exceeds 50% (2). AKI can occur in patients with no prior kidney disease or those with underlying chronic kidney disease. Even a single AKI episode elevates the long-term risk of cardiovascular disease, chronic kidney disease, and all-cause mortality (4, 5). Consequently, AKI imposes substantial medical and socioeconomic burdens on healthcare systems globally.

Early clinical management improves AKI outcomes, yet recent evidence shows 68% of in-hospital AKI cases remain undiagnosed, correlating with 21.1% mortality versus 3.8% in non-AKI patients (6, 7). This diagnostic gap underscores the dual role of clinical guidelines: standardizing care while enhancing AKI recognition. Notably, biochemical criteria identify 3-fold more cases than administrative coding (7), emphasizing the need for systematic guideline implementation. Despite requiring multidisciplinary care, persistent heterogeneity in AKI management across regions highlights the urgency for evidence-based protocols.

To aid clinicians in the timely diagnosis and treatment of AKI, concerted efforts have been undertaken to systematically synthesize evidence-based strategies into clinical practice guidelines (CPGs) through collaborative initiatives by expert panels at national and international levels (8, 9). However, the development of these guidelines has employed disparate methodological frameworks, resulting in substantial heterogeneity across recommendation levels, evidence grading, and other dimensions (10). This variability creates significant implementation challenges, as clinicians lack clarity regarding the methodological rigor and reliability of the evidence underpinning these recommendations.

Therefore, it is imperative to rigorously appraise existing AKI guidelines to delineate methodological limitations and inform iterative refinements for guideline development teams (11). Such appraisal employs validated assessment tools, including the Appraisal of Guidelines for Research and Evaluation II (AGREE II) (12) and Reporting Items for Practice Guidelines in Healthcare (RIGHT) (13). These internationally endorsed frameworks employ standardized criteria to systematically evaluate guideline quality (14). Leveraging these tools, this research aims to evaluate the methodological and reporting quality of current AKI clinical practice guidelines. The findings will assist clinicians in prioritizing evidence-based guidance, identifying areas for improvement, and promoting the development of future clinical guidelines (12, 15).

## Methods

### Literature search strategy

PubMed, NICE (National Institute for Health and Clinical Excellence), and other related databases such as NGC (National Guideline Clearinghouse), GIN (Guidelines International Network), and SIGN (Scottish Intercollegiate Guidelines Network) were systematically searched from January 2012 to January 2023 to collect the clinical guidelines and expert consensus on acute kidney injury. Keywords for search: Acute Kidney Injur*, Acute Renal Injur*, Acute Renal Insufficienc*, Acute Kidney insufficienc*, Acute Kidney Failure*, consensus, clinical practice guideline*, recommendation*. PubMed search strategy: (“Acute Kidney Injur*”[Title/Abstract] OR “Acute Renal Injur*”[Title/Abstract] OR “Acute Renal Insufficienc*”[Title/Abstract] OR “Acute Kidney Insufficienc*”[Title/Abstract] OR “Acute Kidney Failure”[Title/Abstract]) AND (“Practice Guideline”[Publication Type] OR “consensus development conference”[Publication Type] OR “clinical practice guideline”[Title/Abstract] OR recommendation*[Title/Abstract]) AND (“2012/01/01”[Date-Publication]: “2023/01/31”[Date-Publication]) NOT (“editorial”[Publication Type] OR “news”[Publication Type]).

### Inclusion and exclusion criteria

In terms of inclusion criteria, the guidelines had to cover relevant information on the diagnosis or treatment of acute kidney injury. They also had to be available in either Chinese or English and be the latest version of the guide. On the other hand, guidelines were excluded if the full text could not be accessed, if they were repetitively published, or if they were interpretations or summaries of other guidelines.

### Literature search and data extraction

Two investigators independently conducted the literature search and implemented the predefined eligibility criteria. After the initial title/abstract screening of retrieved records, both investigators performed a full-text review of potentially eligible articles for secondary eligibility assessment. Upon finalizing the selection of eligible guidelines and consensus statements, the investigators conducted independent data extraction using a standardized protocol.

The data extraction process involved collecting general information about the evaluated clinical practice guidelines (CPGs), including guideline title, issuing organization, country, publication year, update status, implementation of systematic literature searches (with databases queried, search strategies employed, and search timeframe), funding sources and conflict of interest declarations.

### Quality assessment

To assess the methodological quality of the clinical practice guidelines (CPGs), the AGREE II tool was used (12). Two independent reviewers (R.L. and X.W.) evaluated guideline quality using the AGREE II instrument, which assesses six domains through 23 key items: 1. Scope and Purpose (3 items): Overall objectives, health questions, target population. 2. Stakeholder Involvement (3 items): Development group composition, patient perspectives. 3. Rigor of Development (8 items): Evidence synthesis, recommendation formulation. 4. Clarity of Presentation (3 items): Language accessibility, recommendation specificity. 5. Applicability (4 items): Implementation barriers, resource considerations. 6. Editorial Independence (2 items): Funding transparency, conflict management. Each item was scored on a 7-point Likert scale (1 = strongly disagree to 7 = strongly agree). Domain scores were standardized using:

$$\begin{array}{l} \text{Standardized Score of}\;a\;\text{Domain}\;(\%)\\ =(\begin{array}{l} \text{Obtained Score}-\\ \text{Minimum Possible Score} \end{array})/(\begin{array}{l} \text{Maximum Possible Score}-\\ \text{Minimum Possible Score} \end{array})\times 100\% \end{array}$$

Based on the standardized scores of each domain, the CPGs can be categorized into three recommendation levels:

Level A: CPGs with scores of 70% or higher in 4 or more domains. These guidelines can be directly recommended.

Level B: CPGs that require revision to varying degrees. They have scores of 30% or higher in more than 3 domains and scores of 70% or higher in less than 4 domains.

Level C: CPGs not recommended, as they have scores of 30% or higher in less than 3 domains.

To evaluate the reporting quality of the CPGs, the RIGHT tool was used (13). It assesses seven domains and 22 items, including basic information, background, evidence, recommendations, review and quality assurance, statement and management of funding and conflicts of interest, and others. Each item was scored as “reported” or “not reported.” The overall reporting rate is calculated as: Reporting Rate = Reported Items/Total Applicable Items×100%. The overall reporting rate is divided into three levels: high quality (overall reporting rate of 70%), medium quality (overall reporting rate of 40–70%), and low quality (overall reporting rate less than 40%) (16).

### Consistency inspection

The consistency of scores between the two evaluators using the AGREE II tool was assessed using the intragroup correlation coefficient (ICC). The ICC was calculated using SPSS 25.0. ICC values less than 0.40 indicate poor consistency, 0.40–0.75 indicate general consistency and values equal to or greater than 0.75 indicate high consistency (17, 18).

## Results

### Literature search results

**Figure 1:**
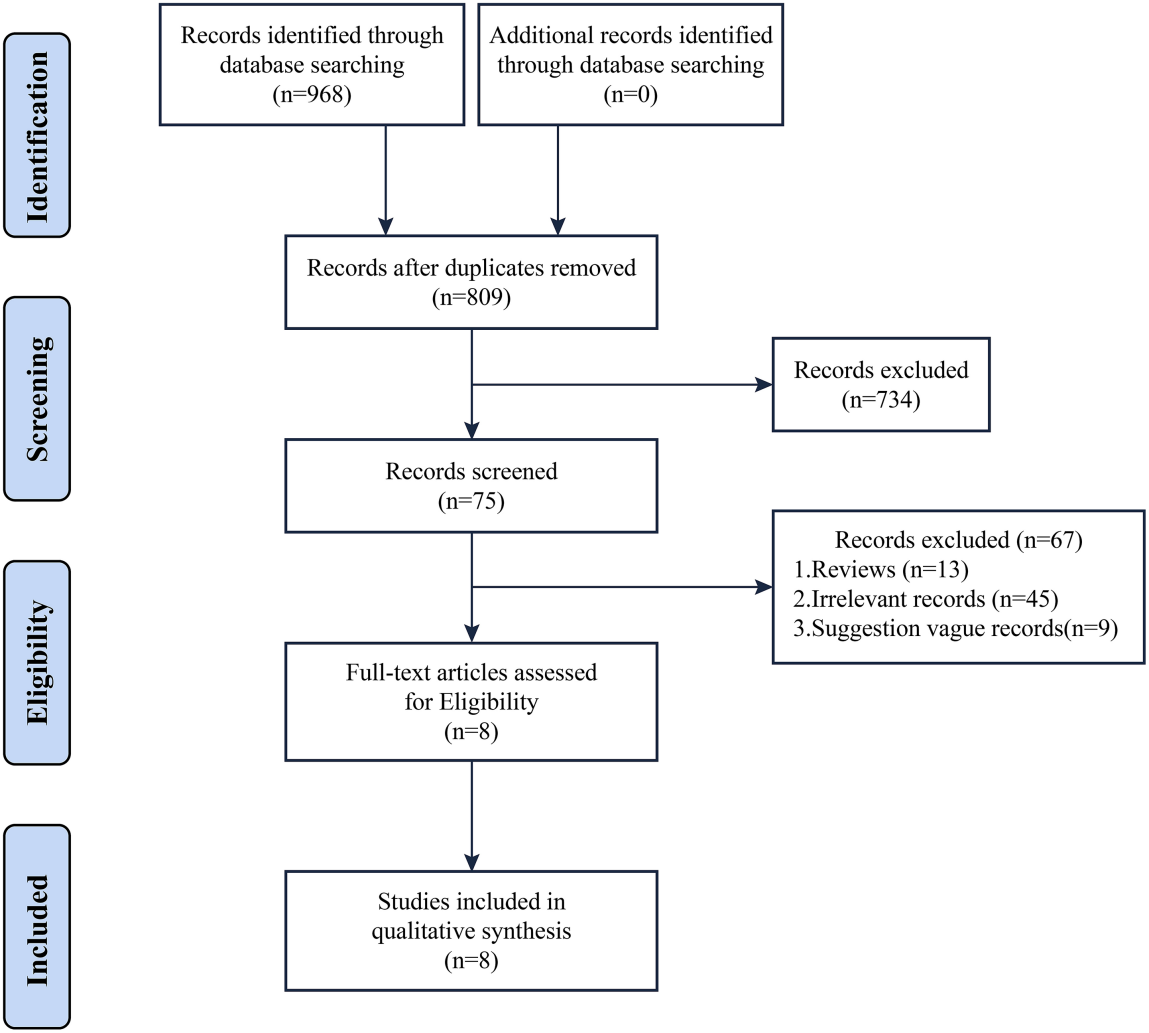
Flowchart of the retrieval strategy.

Based on the literature search results, a total of 968 relevant articles were obtained. After a comprehensive screening process, 8 clinical practice guidelines (CPGs) were finally included in the study (19–26). The diagnosis of AKI in all included guidelines was based on serum creatinine changes and urine output. AKI was defined as any of the following: 1. Increase in serum creatinine (SCR) by ≥ 0.3 mg/dL (≥ 26.5 μmoL/L) within 48 h, or ≥ 1.5 times baseline (known or preset to have occurred within the prior 7 days) 2. urine output (UO) < 0.5 mL/kg/h for 6 h. The literature screening process and results are shown in Figure 1.

### General characteristics of the CPGs

These guidelines were published between 2012 and 2021 and originated from six countries across three continents: two from Asian countries, three from European countries, and one from a North American country (the United States). Among the eight included CPGs, five were first editions.

Four guidelines adopted the GRADE framework for grading the level of evidence and recommendation strength, while three guidelines utilized institution-specific grading systems. One guideline was not graded. The development approach for the guidelines varied, with two being evidence-based, one being consensus-based, and the rest using the Delphi approach (27). All the included CPGs were published in English, and no guidelines in Chinese were found. Four guidelines reported funding sources, which came from societies or expert organizations and were not sponsored by private companies or pharmaceutical companies. Comprehensive methodological details are summarized in Table 1.

**Table 1:** Characteristics of the identified guidelines on the AKI.

Title	Author/organization	Short title	Year	Country applied	Version	Grading system used	Development method	Funding
AKI: prevention, detection, and management (19)	National Institute for Health and Care Excellence	NICE	2019	UK	Second	None	Delphi	Not reported
Nomenclature and diagnostic criteria for AKI 2020 consensus of the Taiwan AKI-task force (20)	Taiwan AKI-TASK Force	TATF	2021	China, Taiwan Province	First	Self-setting	CB	Ministry of Science and Technology, Taiwan
The Japanese Clinical Practice Guideline for AKI 2016 (21)	The Japanese Clinical Practice Guideline for AKI 2016 Committee	JAC	2018	Japan	First	Self-setting	EB	Not reported
KDIGO Clinical Practice Guideline for AKI (22)	Kidney Disease: Improving Global Outcomes (KDIGO) AKI Work Group	KDIGO	2012	Global	First	GRADE	Delphi	Kidney Disease: Improving Global Outcomes (KDIGO) AKI Work Group
Clinical Practice Guideline AKI (23)	The UK Renal Association	RA	2019	UK	third	GRADE	Delphi	The UK Renal Association
Prevention of AKI and protection of renal function in the intensive care unit: update 2017 (24)	European Society of Critical Care	ESCC	2017	Europe	Second	GRADE	Delphi	University of Innsbruck and Medical University of Innsbruck
AKI in the perioperative period and intensive care units (excluding renal replacement therapies) (25)	Multiple organizations and individuals	MOI	2016	France	First	GRADE	Delphi	Not reported
AKI: A Guide to Diagnosis and Management (26)	American Academy of Family Physicians	AFP	2012	US	First	Self-setting	EB	Not reported

GRADE, Grading of Recommendations Assessment, Development and Evaluation; UK, the United Kingdom; US, the United States; CB, consensus-based guideline; EB, evidence-based guideline.

### Methodological and reporting quality of the included guidelines

Table 2 presents the results of the methodological quality assessment for the eight included guidelines using the AGREE II tool. The guidelines collectively demonstrated moderate overall quality. Among the six domains, only two domains exhibited mean scores greater than 50% (Scope and Purpose 50.35%, Clarity of Presentation 77.43%); the rest of the domains scored between 40 and 50%, with Stakeholder Involvement scoring the lowest (41.32%). The recommendation level evaluation of the CPGs summarizes the overall opinion of whether the CPGs should be recommended for clinical use, which shows that only one guideline developed by KDIGO was rated A (strong recommendation), and the remaining seven guidelines were rated B (general recommendation).

**Table 2:** AGREE II outcomes.

AGREE II scores of AKI guidelines								
Guideline	Scope and purpose	Stakeholder involvement	Rigour of development	Clarity of presentation	Applicability	Editorial independence	Mean ± SD	Grade
NICE	75.00	27.78	21.88	75.00	47.92	0.00	41.26 ± 27.63	B
TATF	52.78	50.00	47.92	80.56	50.00	62.50	57.29 ± 11.42	B
JAC	50.00	27.78	50.00	86.11	50.00	58.33	53.70 ± 17.25	B
KDIGO	44.44	72.22	62.50	83.33	72.92	91.67	71.18 ± 15.06	A
RA	44.44	55.55	72.92	91.67	56.25	45.83	61.11 ± 16.53	B
ESCC	55.55	44.44	50.00	72.22	37.50	58.33	53.01 ± 11.01	B
MOI	38.89	33.33	33.33	50.00	25.00	41.67	37.04 ± 7.80	B
AFP	41.67	19.44	51.04	80.56	39.58	25.00	42.88 ± 19.87	B
Mean	50.35	41.32	48.70	77.43	47.40	47.92		

The reporting quality of the guidelines was evaluated using the RIGHT tool (Table 3). The overall reporting completeness across the seven RIGHT domains was moderate (average overall reporting rate: 57.66 ± 15.20%). Among the seven fields, the average reporting rate of four fields exceeded 50%, while notable deficiencies were observed in Review and quality assurance (25.00%), Backgrounds (39.06%), and Recommendations (46.23%). According to pre-established grading criteria, the reporting rate of guidelines developed by RA and KDIGO was greater than 70.00% (high quality), and the reporting rate of the remaining guidelines was between 40.00 and 70.00% (medium quality).

**Table 3:** RIGHT summary.

	Basic information	Backgrounds	Evidence	Recommendations	Review and quality assurance	Funding declaration and management	Other information	Mean
NICE	83.33	50.00	40.00	28.57	0.00	50.00	100.00	50.27
TATF	83.33	37.50	60.00	42.86	50.00	25.00	100.00	56.96
JAC	66.67	0.00	40.00	57.14	0.00	0.00	66.67	32.93
KDIGO	83.33	75.00	80.00	71.43	100.00	75.00	100.00	83.54
RA	83.33	62.50	80.00	57.14	50.00	75.00	100.00	72.57
ESCC	83.33	37.50	100.00	42.86	0.00	50.00	100.00	59.10
MOI	100.00	25.00	80.00	42.86	0.00	100.00	100.00	63.98
AFP	83.33	25.00	40.00	28.57	0.00	50.00	66.67	41.94
Mean ± SD	83.33 ± 8.33	39.06 ± 22.04	65.00 ± 21.79	46.23 ± 13.83	25.00 ± 35.36	53.13 ± 29.15	91.67 ± 14.43	57.66 ± 15.20

When assessing the methodological quality and reporting of AKI clinical guidelines, no single guideline demonstrated comprehensive excellence across all assessment domains. All guides have strengths, but weaknesses should be addressed in the future. These observations should be interpreted not as critiques, but as actionable insights to guide iterative refinements in guideline development processes. It is imperative to emphasize that this study is an analysis of the methodology and reporting quality included and does not attest to the accuracy of the recommendations made. Future research should systematically compare inter-guideline recommendation concordance to establish consensus on optimal AKI management strategies.

### Summary of AKI diagnosis and treatment recommendations

Except for the diagnostic guidelines of the TATF, all other guidelines addressed the treatment and management of acute kidney injury (AKI). The guidelines exhibited substantial heterogeneity in their target patient populations. The ESCC guidelines emphasized AKI prevention and renal function protection in ICU patients, while the MOI guidelines emphasized early detection and prevention of AKI in perioperative patients. The KDIGO, RA, NICE, JAC, and AFP guidelines were more broadly applicable, providing tailored therapeutic regimens for complex presentations such as hypovolemic shock and hypercoagulable states. The recommendations typically encompassed medication, supportive care, and renal replacement therapy. Pharmacotherapeutic strategies centered on evidence-based diuretic administration. Supportive care and RRT emerged as primary therapeutic priorities across the majority of guidelines. Supportive care encompassed appropriate protein intake, nutritional support, and maintenance of water and electrolyte balance. Renal replacement therapy encompassed indications for continuous renal replacement therapy, hemodialysis, or peritoneal therapy, alongside anticoagulation measures during renal replacement therapy.

## Discussions

### General characteristics of CPGs

Our analysis of 8 contemporary AKI guidelines (2013–2022) revealed two critical gaps in addressing aging populations: 1. Methodological Disparities: While 62% (5/8) adopted GRADE evidence grading (e.g., KDIGO, ESCC), consensus-based guidelines like TATF failed to specify age-adjusted diagnostic thresholds. This is particularly problematic given older adults (≥65 years) comprise 60–65% of AKI hospitalizations (28). 2. Geriatric Adaptation Deficits: Only 25% (2/8) provided age-stratified recommendations despite creatinine baseline variability increasing with age. To address demographic shifts, future guidelines should use age-corrected equations to define baseline creatinine and clinical frailty scores that can be considered for inclusion in AKI staging in older patients.

### Methodological limitations and future directions

Our analysis revealed critical shortcomings in current AKI guidelines through a rigorous AGREE II evaluation (inter-rater ICC ≥ 79.7%). Four domains scored below 50%, with Stakeholder Involvement (41.32%) and Applicability (47.40%) representing the most pressing concerns. The systemic exclusion of patient perspectives, particularly vulnerable populations like older adults, from guideline development processes directly compromises clinical implementation. This oversight manifests as recommendations misaligned with real-world patient needs, exemplified by the underrepresentation of age-related considerations in diagnostic thresholds and therapeutic strategies for geriatric AKI populations (29). Geriatricians and nephrologists should cooperate for the management of these patients (30).

The parallel deficiency in Applicability reflects inadequate adaptation to evolving demographic realities. With older adults constituting over 65% of hospitalized AKI cases globally, future guidelines must explicitly address age-specific challenges, including comorbid polypharmacy management (31), altered renal reserve, and frailty-adjusted treatment goals (32). Practical implementation requires concurrent development of tiered support tools, such as age-stratified dosing calculators and comorbidity-specific care pathways, to bridge the gap between evidence synthesis and bedside application.

Integrating geriatric nephrology specialists and patient advocacy groups into guideline panels improves both stakeholder representation and clinical relevance. While resource constraints persist, pilot programs demonstrate that cost-effective strategies like virtual patient engagement platforms and adaptive Delphi methods can overcome traditional barriers to inclusive guideline development (33). These innovations, coupled with mandatory reporting of age-disaggregated outcomes in AKI research, will be essential for creating guidelines that reflect our aging global population.

### Reporting limitations and future directions

Our evaluation of AKI guidelines revealed gaps in standardized reporting despite overall satisfactory methodological quality. While most guidelines demonstrated strong adherence to structural formatting (91.67% in “Other Information”), significant deficiencies emerged in Background Review and Quality Assurance, with average reporting rates of only 39.06 and 25.00%, respectively. These findings align with recent evidence highlighting persistent underrecognition of AKI in clinical practice, particularly among older adults and surgical populations (34, 35). This indicates that there is room for improvement in these crucial areas of guideline development.

The observed shortcomings in the backgrounds of CPGS may partially explain why current guidelines inadequately address the unique diagnostic and therapeutic challenges in different populations, especially among older adults. Older adults frequently present with atypical AKI manifestations, polypharmacy-related risks, and complex comorbidity profiles that existing classification systems often fail to capture (36). For instance, our analysis found few guidelines providing age-adjusted thresholds for serum creatinine changes or explicit recommendations for medication reconciliation in frail elderly patients. To address these limitations, future guideline development should prioritize one key enhancement: Stricter adherence to reporting standards for evidence synthesis processes, particularly regarding the inclusion of geriatric-specific outcomes data and real-world effectiveness studies. Strengthening quality assurance protocols through prospective validation in diverse age groups could improve guideline applicability across care settings.

**Figure 2:**
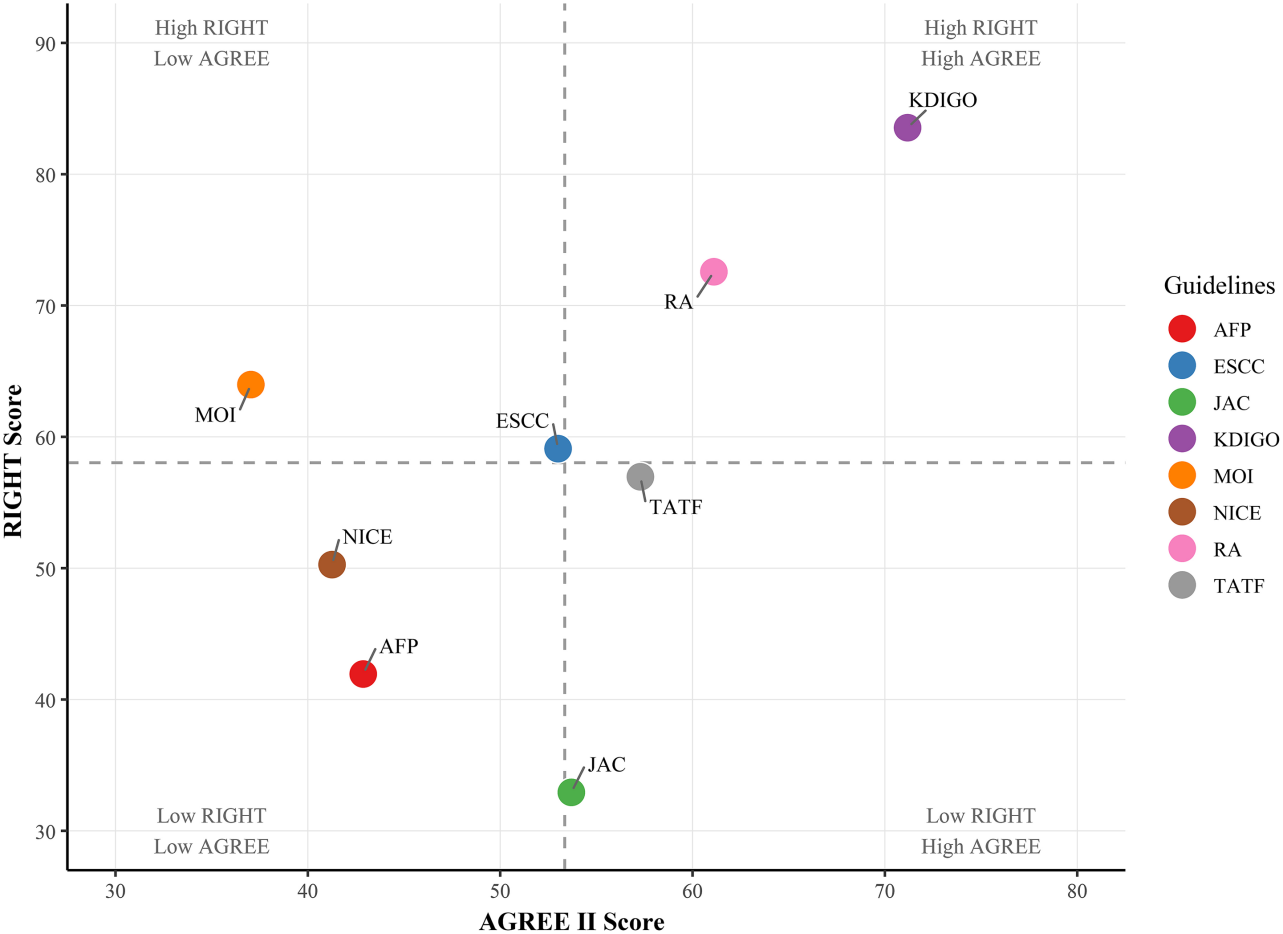
A scatter plot describes guidelines for the consistency and accuracy of the score.

Our analysis revealed substantial variability in the implementation of peer review and quality assurance processes among clinical practice guidelines (CPGs). Only KDIGO explicitly described both procedures, while others either omitted reporting or lacked transparency in methodological rigor. The superiority of KDIGO’s approach is further supported by its highest AGREE II and RIGHT scores in our evaluation (Figure 2), followed by RA guidelines. This underscores the critical role of structured review systems in enhancing guideline reliability.

A significant limitation of this study lies in the fact that any assessment tool employed for guidelines relies solely on the information reported by their developers, potentially obfuscating the actual intricacies of the development process. Despite the utilization of standardized tools by two independently trained evaluators, subjective bias remains an unavoidable aspect of the evaluation process. Additionally, the study’s scope is confined to guidelines published after 2012, and the language restriction to Chinese and English may introduce selection bias, thereby limiting the generalizability of the findings.

## Conclusion

Our appraisal identifies the 2012 KDIGO guideline as the highest-quality AKI guidance framework currently available, which has driven global standardization of diagnostic criteria and management protocols. Guideline development has significantly increased clinical awareness of AKI. However, our analysis reveals critical gaps in practical applicability and transparency, particularly regarding implementation strategies tailored to different populations, such as aging populations. Future iterations must prioritize demographic inclusivity, with explicit recommendations addressing the diagnostic challenges and management complexities of AKI among different populations.

## Data Availability

The original contributions presented in the study are included in the article/supplementary material, further inquiries can be directed to the corresponding author.

## Supplementary material

SUPPLEMENTARY TABLE S1 Full methodological assessment data for the eight included clinical practice guidelines.
