# Supplementary material for: Clinical practice guidelines for acute kidney injury: a systematic review of the methodological quality
Source: Front Med (Lausanne). 2025 Jul 24;12:1567359. doi: 10.3389/fmed.2025.1567359 (PMC12328445; doi:10.3389/fmed.2025.1567359)
Supplement: SUPPLEMENTARY TABLE S1 — Full methodological assessment data for the eight included clinical practice guidelines. [file Table_1.DOCX]

**The below tables are ONLlNE Supplementary materials**

**Table S1 AKI medication**

| Short tittle | Number of recommendations | Recommendations | | | Evidence level | Recommended strength |
| --- | --- | --- | --- | --- | --- | --- |
|  |  | Original text of the guides | Indications | Recommended treatment options |  |  |
| NICE | 1 | Consider loop diuretics for treating fluid overload or oedema while: an adult, child or young person is awaiting renal replacement therapy ; renal function is recovering in an adult, child or young person not receiving renal replacement therapy. | Patients with fluid overload or edema. | loop diuretics | Unreported | |
| TATF | 0 | none | / | / | / | / |
| JAC | 2 | We do not recommend loop diuretics for the prevention of AKI. We also suggest that loop diuretics should not be administered for the treatment of AKI, except to correct fluid overload. | Patients with fluid overload or edema | loop diuretic | C | 2 |
|  |  | Recommendation: Nafamostat mesilate may be considered for patients with a high risk of bleeding. For patients with active bleeding, blood purification without the use of anticoagulants may also be considered. | Patients at high risk of bleeding | Nafamostat mesylate | C | Unreported |
| KDIGO | 3 | We recommend the use of vasopressors in conjunction with fluids in patients with vasomotor shock with, or at risk for, AKI. | Patients at high risk of bleeding | Vasopressors with fluids | C | 1 |
|  |  | We suggest not using diuretics to treat AKI, except in the management of volume overload. | Patients with vasomotor shock with,or at risk for, AKI. | Diuretics | C | 2 |
|  |  | We suggest that a single dose of theophylline may be given in neonates with severe perinatal asphyxia,who are at high risk of AKI. | Patients with fluid overload or edema. | Single dose of theophylline | B | 2 |
| RA | 1 | We recommend that optimisation of haemodynamic status using appropriate fluid therapy and administration of vasopressors and/or inotropes as appropriate. | AKI patients or at high risk of developing AKI | Fluid therapy and administration of vasopressors and/or inotropes | B | 1 |
| ESCC | 4 | We suggest using diuretics to control or avoid fluid overload in patients that are diuretic-responsive. | Patients with fluid overload or edema. | Diuretics | D | 2 |
|  |  | We recommend titrating vasopressors to a mean arterial pressure (MAP) of 65–70 mmHg rather than a higher MAP target (80–85 mmHg) in patients with septic shock. However, for patients with chronic hypertension we recommend aiming for a higher target (80–85 mmHg) for renal protection in septic shock. | patients with septic shock. | Vasopressors to a mean arterial pressure (MAP) of 65–70 mmHg | B | 1 |
|  |  |  | patients with chronic hypertension and septic shock. | Vasopressors to a mean arterial pressure (MAP) of 80–85 mmHg | C | 1 |
|  |  | We recommend lowering systolic pressure to 140–190 mmHg rather than to 110–139 mmHg in patients with acute cerebral haemorrhage with severe admission hypertension. | patients with acute cerebral haemorrhage with severe admission hypertension | lowering systolic pressure to 140–190 mmHg | C | 1 |
|  |  | If vasopressors are needed for treatment of hypotension, we recommend norepinephrine (along with correction of hypovolaemia) as the first-choice vasopres sor to protect kidney function and suggest vasopressin in patients with vasoplegic shock after cardiac surgery. | patients with hypotension  vasoplegic shock after cardiac surgery | norepinephrine | B | 1 |
|  |  |  |  | vasopressin | C | 2 |
| MOI | 1 | We recommend not using diuretics in order to prevent or treat AKI; we suggest using them for treating fluid overload. | Patients with fluid overload or edema. | Diuretics | Unreport | 1 |
| AFP | 2 | Diuretics do not improve morbidity, mortality, or renal outcomes, and should not be used to prevent or treat acute kidney injury in the absence of volume overload. | Patients with fluid overload or edema. | Diuretics | A | Unreported |
|  |  | Consider therapy with immunosuppressive agents (e.g., cyclophosphamide, prednisone) in patients with rapidly progressive glomerulonephritis. | patients with rapidly progressive glomerulonephritis | immunosuppressive agents | C | unreported |

**Table S2 Supportive care**

**Table 4**（continue）

| Short tittle | Number of recommendations | Recommendations | | | Evidence level | Recomme-nded strength |
| --- | --- | --- | --- | --- | --- | --- |
|  |  | Original text of the guides | Indications | Recommended treatment options |  |  |
| NICE | 0 | none | / | / | / | / |
| TATF | 0 | none | / | / | / | / |
| JAC | 1 | We suggest that the administration of calorie and protein as nutritional support for AKI treatment be tailored to the severity and the underlying disease. For severe AKI, we recommend enteral nutrition whenever possible. Unless there is an advanced electrolyte imbalance, strict protein restriction is not necessary. | AKI patients | calorie and protein as nutritional support | D | 2 |
|  |  |  | severe AKI | enteral nutrition |  |  |
|  |  |  | AKI patients with advanced electrolyte imbalance | strict protein restriction |  |  |
| KDIGO | 5 | In the absence of hemorrhagic shock, we suggest using isotonic crystalloids rather than colloids (albumin or starches) as initial management for expansion of intravascular volume in patients at risk for AKI or with AKI. | AKI patients without hemorrhagic shock | isotonic crystalloids | B | 2 |
|  |  | In critically ill patients, we suggest insulin therapy targeting plasma glucose 110-149 mg/dl(6.1-8.3 mmol/l). | Critically patients | insulin therapy targeting plasma glucose 110-149 mg/dl | C | 2 |
|  |  | We suggest achieving a total energy intake of 20– 30 kcal/kg/d in patients with any stage of AKI. | AKI patients | energy intake of 20– 30 kcal/kg/d | C | 2 |
|  |  | We suggest administering 0.8-1.0 g/kg/d of protein in noncatabolic AKI patients without need for dialysis, 1.0-1.5 g/kg/d with AKI on RRT, and up to a maximum of 1.7 g/kg/d in patients on continuous renal replacement therapy(CRRT) and in hypercatabolic patients. | Noncatabolic AKI patients without need for dialysis | 0.8-1.0 g/kg/d of protein | D | 2 |
|  |  |  | patients with AKI on RRT | 1.0-1.5 g/kg/d of protein |  |  |
|  |  |  | patients on continuous renal replacement therapy (CRRT) and in hypercatabolic patients | maximum of 1.7 g/kg/d of protein |  |  |
|  |  | We suggest providing nutrition preferentially via the enteral route in patients with AKI. | AKI patients | Enteral nutrition | C | 2 |
| RA | 2 | We recommend that adult and paediatric patients identified as being at risk of developing AKI due to rhabdomyolysis, and who are not volume overloaded, should receive prompt intravenous volume expansion in order to achieve a high urinary flow rate. | adult and paediatric patients identified as being at risk of developing AKI due to rhabdomyolysis, and who are not volume overloaded | intravenous volume expansion | B | 1 |
|  |  | We recommend that veno-venous access is used for acute renal replacement therapy. | patients need acute renal replacement therapy | veno-venous access | A | 1 |
| ESCC | 6 | We recommend controlled fluid resuscitation in volume depletion, while, however, avoiding volume overload. | hypovolemic patients | controlled fluid resuscitation | C | 1 |
|  |  | We recommend correction of hypovolaemia/dehydration using isotonic crystalloids in patients receiving intravascular contrast media. | patients receiving intravascular contrast media | isotonic crystalloids | B | 1 |
|  |  | We suggest the use of balanced crystalloids for large volume resuscitation. | AKI patients need large volume resuscitation. | balanced crystalloids | C | 2 |
|  |  | We suggest using human serum albumin if a colloid is deemed necessary for the treatment of patients with septic shock. | patients with septic shock | human serum albumin | C | 2 |
|  |  | We suggest targeting a blood glucose level at least below 180 mg/dL (10 mmol/l) for the prevention of hyperglycaemic kidney damage in the general ICU population. | general ICU population | targeting a blood glucose level at least below 180 mg/dL (10 mmol/l) | B | 2 |
|  |  | We suggest that all patients with or at risk of acute kidney injury have adequate nutritional support preferably through the enteral route. | all patients with or at risk of acute kidney injury | adequte enteral nutrition | unreported | BPS |
| MOI | 2 | We suggest following the same nutritional strategy rules in critically ill patients whether or not they have AKI (without renal replacement therapy). | critically ill patients | without RRT：provides 20–30 kcal/kg/day of energy and 1.5 g/kg/day of protein. | unreported | Grade 2+ |
|  |  |  |  | on RRT：increase protein supply | unreported | Grade 2+ |
|  |  | We suggest adapting protein intake according to the age of children with AKI. | children with AKI. | protein intake according to the age：0-2 years old 2-3 g/kg/day; 2-13 years old 1.5-2 g/kg/day; 13 years old and above 1.5 g/kg/day. | unreported | Grade 2+ |
| AFP | 1 | Adequate fluid balance should be maintained in patients with acute kidney injury by using isotonic solutions (e.g., normal saline) instead of hyperoncotic solutions (e.g., dextrans, hydroxyethyl starch, albumin). | AKI patients | isotonic solutions (e.g., normal saline) to maintain fluid balance | A | unreported |

**Table S3** AKI medication

| Short tittle | Number of recommendations | Recommendations | | | | | Evidence level | Recommended strength |
| --- | --- | --- | --- | --- | --- | --- | --- | --- |
|  |  | Original text of the guides | | Indications | | Recommended treatment options |  |  |
| NICE | 1 | Refer adults, children and young people immediately for renal replacement therapy if any of the following are not responding to medical management: hyperkalaemia；metabolic acidosis symptoms or complications of uraemia (for example, pericarditis or encephalopathy)；fluid overload；pulmonary oedema | | Occurrence of any of the following: hyperkalemia, metabolic acidosis, uremia, fluid overload, pulmonary edema and not responding to medical therapy | | RRT | unreported | |
| TATF | 0 | none | | / | | / | / | / |
| JAC | 2 | Recommendation: In hemodynamically stable patients, blood purification may be performed either continuously or intermittently. In hemodynamically unstable patients, continuous blood purification is preferable. | | hemodynamically stable patients | | continuous or intermittent blood purification | B | 2 |
|  |  |  |  | hemodynamically unstable patients | | continuous blood purification | C | unreported |
|  |  | Recommendation: Nafamostat mesilate may be considered for patients with a high risk of bleeding. For patients with active bleeding, blood purification without the use of anticoagulants may also be considered. | | patients with active bleeding | | blood purification without anticoagulants | C | unreported |
| KDIGO  **Table5**（continue） | 8 | Initiate RRT emergently when life-threatening changes in fluid, electrolyte, and acid-base balance exist. | | life-threatening changes in fluid, electrolyte, and acid-base balance | | Initiate RRT emergently | unreported | |
|  |  | We recommend using anticoagulation during RRT in AKI if a patient does not have an increased bleeding risk or impaired coagulation and is not already receiving systemic anticoagulation. | | patient does not have an increased bleeding risk or impaired coagulation and is not already receiving systemic anticoagulation. | | using anticoagulation during RRT | B | 1 |
|  |  | For patients without an increased bleeding risk or impaired coagulation and not already receiving effective systemic anticoagulation, we suggest the following: | For anticoagulation in intermittent RRT ,we recommend using either unfractionated or low-molecular-weight heparin,rather than other anticoagulants. | atients without an increased bleeding risk or impaired coagulation and not already receiving effective systemic anticoagulation | intermittent RRT | either unfractionated or low-molecular-weight heparin | C | 1 |
|  |  |  | For anticoagulation in CRRT, we suggest using regional citrate anticoagulation rather than heparin in patients who do not have contraindications for citrate. |  | in CRRT, patients do not have contraindications for citrate. | regional citrate anticoagulation | B | 2 |
|  |  |  | For anticoagulation during CRRT in patients who have contraindications for citrate, we suggest using either unfractionated or low-molecular-weight heparin, rather than other anticoagulants. |  | in CRRT, patients have contraindications for citrate. | either unfractionated or low-molecular-weight heparin | C | 2 |
|  |  | For patients with increased bleeding risk who are not receiving anticoagulation, we suggest the following for anticoagulation during RRT: We suggest using regional citrate anticoagulation, rather than no anticoagulation, during CRRT in a patient without contraindications for citrate. | | patients with increased bleeding risk who are not receiving anticoagulation | | regional citrate anticoagulation | C | 2 |
|  |  | In a patient with heparin-induced thrombocytopenia(HIT), all heparin must be stopped and we recommend using direct thrombin inhibitors (such as argatroban) or Factor Xa inhibitors (such as danaparoid or fondaparinux) rather than other or no anticoagulation during RRT. | | patient with heparin-induced thrombocytopenia(HIT) | | using direct thrombin inhibitors (such as argatroban) or Factor Xa inhibitors (such as danaparoid or fondaparinux) during RRT. | A | 1 |
|  |  | In a patient with HIT who does not have severe liver failure, we suggest using argatroban rather than other thrombin or Factor Xa inhibitors during RRT. | | patients with HIT but does not have severe liver failure | | argatroban during RRT | C | 2 |
|  |  | We suggest using CRRT, rather than standard intermittent RRT, for hemodynamically unstable patients. | | hemodynamically unstable patients | | CRRT | B | 2 |
|  |  | We suggest using CRRT, rather than intermittent RRT, for AKI patients with acute brain injury or other causes of increased intracranial pressure or generalized brain edema. | | AKI patients with acute brain injury | | CRRT | B | 2 |
| RA | 8 | We recommend that acute renal replacement therapy (RRT) should be considered for patients with progressive or severe AKI, unless a decision has been made not to escalate therapy. | | patients with progressive or severe AKI | | RRT | B | 1 |
|  |  | We recommend that peritoneal dialysis may be considered as an alternative to extracorporeal treatments in paediatric patients. | | paediatric patients | | peritoneal dialysis as an alternative to extracorporeal treatments | B | 1 |
|  |  | Continuous RRT should preferably be offered to patients who are haemodynamically unstable or have acute brain injury or cerebral oedema. | | patients who are haemodynamically unstable or have acute brain injury or cerebral oedema | | Continuous RRT | B | 2 |
|  |  | We recommend that for anticoagulation in CRRT, regional citrate anticoagulation should be the first line choice. When citrate is contraindicated or not available, unfractionated heparin or epoprostenol should be considered. | | anticoagulation in CRRT | | regional citrate anticoagulation | B | 1 |
|  |  |  |  | citrate is contraindicated or not available | | heparin or epoprostenol |  |  |
|  |  | We recommend that for anticoagulation in acute intermittent RRT, unfractionated heparin or low molecular weight heparin should be used as the first line anticoagulant. | | in acute intermittent RRT | | unfractionated heparin or low molecular weight heparin | C | 1 |
|  |  | We recommend that patients with AKI treated by CRRT should receive treatment doses equivalent to post dilution ultrafiltration rates of 25 ml/kg/hr. | | patients with AKI treated by CRRT | | treatment doses equivalent to post dilution ultrafiltration rates of 25 ml/kg/hr | A | 1 |
|  |  | We recommend that consideration should be given to the risk of dialysis disequilibrium syndrome in patients initiating intermittent haemodialysis with a high serum urea and that a lower intensity first dialysis should be prescribed for patients at risk. | | patients at high risk of Dialysis Imbalance Syndrome. | | a lower intensity first dialysis | B | 1 |
|  |  | We recommend that the decision to start RRT in patients with AKI should be based on fluid, electrolyte and metabolic status of each individual patient. It should be started before the onset of life threatening complications of AKI unless a decision has been made that escalation of therapy is not appropriate. | | before the onset of life threatening complications of AKI | | RRT | C | 1 |
| ESCC | 0 | none | | / | | / | / | / |
| MOI | 0 | none | | / | | / | / | / |
| AFP | 0 | none | | / | | / | / | / |
